# Aspirin is associated with low oral pH levels and antacid helps to increase oral pH

**DOI:** 10.1186/s13104-018-3247-3

**Published:** 2018-02-20

**Authors:** Dileepa Senajith Ediriweera, Nuwani Dilina, Vipula Saparamadu, Inoka Fernando, Buddhika Kurukulasuriya, Deepika Fernando, Janakie Kurera

**Affiliations:** 1 Centre for Health Informatics, Biostatistics and Epidemiology, Faculty of Medicine, University of Kelaniya, Ragama, Sri Lanka; 2 Ministry of Health, Colombo, Sri Lanka

**Keywords:** Aspirin, Oral pH, Non-communicable disease, Primary and secondary prevention of non-communicable diseases

## Abstract

**Objective:**

Aspirin is a commonly used medicine for primary and secondary prevention of cardiovascular diseases. It is an acidic medicine associated with gastric irritation and acid reflux, which in turn can lead to low oral pH levels. Therefore, it is important to understand the association between aspirin and oral pH levels in order to achieve an optimum oral health condition among patients who take aspirin on prescription.

**Results:**

Out of 373 patients, 162 (44%) were males and 245 (66%) were on aspirin. 71% of aspirin taking patients and 29% of non-aspirin taking patients had oral pH less than 6.5 (P < 0.01). Aspirin showed a significant association with low oral pH levels (odds ratio = 1.91, 95% CI 1.23–2.99, P < 0.01). 78 patients were given antacids and followed up for 4 weeks, 63 of them (81%) showed an improvement in oral pH and the improvement was marked in the group who had oral pH between 5.5–6.0 compared to the group who had oral pH between 6.0–6.5 (P = 0.03). The results show that aspirin therapy is associated with low oral pH and administration of an antacid with aspirin helps to increase the oral pH level.

## Introduction

Cardiovascular diseases (CVDs) have been identified as the number one cause of death in the world and they cause 31% of all global deaths [1]. Long term low dose aspirin (acetylsalicylic acid) is used for primary and secondary prevention of CVDs and aspirin has become one of the most widely prescribed drugs worldwide [2]. Aspirin is considered as an essential medicine in Sri Lanka and is widely prescribed through the tax revenue funded state healthcare service which provides free healthcare service to the public [3].

Oral pH level mainly depends on saliva and the unstimulated saliva has pH of 6.8 on average. Salivary pH increases upon stimulation and higher oral pH levels aid to buffer acids, suppress aciduric bacteria and remineralize the of enamel [4]. Although there is no predefined critical oral pH level for dental erosions, studies have shown that long term exposure to low pH levels lead to enamel demineralization [5, 6]. Aspirin itself is an acidic medicine and causes gastric irritation and regurgitation which can lead to low oral pH levels [7]. It has been shown that the aspirin taking rheumatoid arthritis patients experience dental erosions [4, 8] and it is believed that these dental erosions are caused by the topical effect of chewed form of aspirin [9].

However, there is a scarcity of literature on the effect of aspirin on oral pH levels. Therefore, we undertook this study to assess the effect of swallowed aspirin on the oral pH levels of the patients who are attending the regular medical clinics.

## Main text

### Methods

This was a multicenter study done at the Family Medicine Clinic of District General Hospital, Negombo and Central Dispensary, Uswetakeiyawa in Gampaha District, Sri Lanka. Patients attending these medical clinics for their regular follow up visits to obtain treatment for hypertension, ischaemic heart disease and cerebrovascular disease were considered for the study. These patients received relevant medicine including aspirin, antihypertensives, nitrates, statins and antacids for their disease conditions. The systematic sampling method was used to recruit study participants where every 10th patient attending the clinics on each day was considered as eligible for the study. Patients who had taken acidic food or alkali food were excluded and those who had a traditional rice based Sri Lankan breakfasts were selected to minimize the effect of food on oral pH levels. Diabetic patients (due to acidic oral conditions), patients with heart failure (due to acid base imbalances), regular beetle chewers (due to dynamic oral pH alterations) and patients on other anti-thrombotic agents were also excluded. All government hospitals and clinics in Sri Lanka receive almost the same medicine brands at a given time period. Patients taking aspirin brands not prescribed by the government health service were also excluded to ensure that all participants received the same medicines.

An interviewer administrated questionnaire was used to collect data on age, gender, past medical history, drug history and duration of aspirin intake. Since the intra-oral pH mainly depends on the salivary pH [10], we measured salivary pH using litmus pH papers with a pH range from 5 to 8. Salivary samples were obtained between 8 a.m. to 12 p.m. to minimize diurnal variation in salivary samples and saliva was obtained at least 2 h after the last meal [11]. Participants were given distilled water to rinse their mouth and saliva was collected 10 min later. Saliva was collected with minimum oral movements using resting drooling method where patients were asked to sit comfortably and tilt their heads slightly down to pool saliva in the mouth and expectorate. First saliva expectoration was discarded to minimize contamination of saliva. The second saliva sample was used to measure oral pH where patients were asked to drool saliva into sterile containers with a pH paper inside. After allowing 2 min for the pH paper to immerse in saliva, the color of the pH paper was matched to the color chart and pH reading was recorded. Among the aspirin taking patients, those who had low oral pH levels (i.e. 6.5 or less) and not on antacids were considered for the follow up. Among them, 78 who gave consent were prescribed famotidine 20 mg twice a day for 4 weeks and oral pH was re-measured.

For the data analysis, patients were categorized into two groups based on their oral pH; pH readings less than 6.5 were considered as “low oral pH” and the rest were considered as “normal or high oral pH”. Initially, individual variable analysis was carried out using simple linear logistic regression and variables significant at P = 0.2 level were then considered for model fitting. Hosmer and Lemeshow goodness of fit test was used to evaluate the goodness of the fitted models [12, 13]. Since overdispersion was noted in the final fitted model, generalized linear model with quasibinomial distribution was adopted to obtain parameter estimates [14]. The effect of study variables was evaluated using odds ratio, confident interval and P value. Analysis was done with R Programming Language version 3.2.3 [15].

Ethical clearance for the study was obtained from Faculty of Medicine, University of Kelaniya, Sri Lanka and written informed consent was obtained from all the participants before they participated in the study. All the interviews and salivary pH measurements were done by the medical officers and privacy of patients were ensured during the interviews and oral pH examinations.

### Results

Three hundred and seventy-three patients were included in the study. There were 162 (44.1%) males and the median (interquartile range) age of the study population was 63 (56–68) years. The majority of patients had either cardiovascular accidents or hypertension and more than one-third of patients had symptoms and signs of gastritis. There were 245 (65.7%) patients on aspirin (75–150 mg daily) and their median (interquartile range) duration of aspirin prescription was 60 (36–96) months. The characteristics of the study sample are given in Table 1.

**Table 1:** Study participants’ characteristics

	Number (%) or median (IQR)
Demography	
Males	162 (44.1%)
Age (years)	63 (56–68)
Diagnosis	
HTN along	126 (34.2%)
IHD along	73 (19.8%)
CVA along	145 (39.4%)
Either HTN or IHD or CVA	24 (6.6%)
Current medications	
Aspirin	245 (65.7%)
Antihypertensives	230 (61.7%)
Antacids	136 (36.5%)
Nitrates	160 (42.9%)
Statins	244 (65.8%)
Other relevant signs and symptoms	
Gastritis	137 (36.7%)
Sinusitis	132 (35.4%)

*HTN* hypertension, *IHD* ischaemic heart disease, *CVA* cerebrovascular accidents

Among the study population, 237 (63.9%) had oral pH levels less than 6.5 and 134 (36.1%) had oral pH more than 6.5. Among the patients who had oral pH below 6.5, 70.9% (168 out of 237) took aspirin and 29.1% (69 out of 237) did not take aspirin (Pearson’s Chi square test, χ^2^ = 7.8, degree of freedom = 1, P < 0.01). Further, 89 (24.0% of total) showed oral pH less than 6.0, and 85.6% (76 out of 89) of them received aspirin and 14.6% (13 of 89) did not receive aspirin (Pearson’s Chi square test, χ^2^ = 19.4, degree of freedom = 1, P < 0.01). The majority of patients who showed a low oral pH level (i.e. < 6.5) had signs and symptoms of gastritis and sinusitis and these patients received the highest percentages of aspirin, antihypertensives, statins, nitrates and antacids as well. Distribution of signs, symptoms and medicines among the patients with respect to oral pH levels are given in Table 2.

**Table 2:** Comparison of sample characteristics in patients who have a low oral pH versus others

	Oral pH < 6.5 (n = 237)	Oral pH ≥ 6.5 (n = 134)
Number of patients with gastritis (%)	77 (56.2%)	60 (43.8%)
Number of patients with sinusitis (%)	72 (54.6%)	60 (45.4%)
Number of patients taking aspirin (%)	168 (69.1%)	75 (30.9%)
Number of patients taking antihypertensives (%)	150 (65.8%)	78 (34.2%)
Number of patients taking statins (%)	163 (67.4%)	79 (32.6%)
Number of patients taking nitrates (%)	99 (61.9%)	61 (38.1%)
Number of patients taking antacids (%)	75 (55.1%)	61 (44.9%)

Among the aspirin taking 245 patients, 16 (6.5%) had signs and symptoms of gastritis and 10 (4.1%) were on an antacid. Out of 128 patients who were not on aspirin, 121 (94.5%) showed signs and symptoms of gastritis and 126 (98.4%) were on antacids. Six (2.4%) of the aspirin taking patients and 126 (98.4%) of non-aspirin taking patients showed signs and symptoms of sinusitis.

Individual variable analysis revealed that aspirin, antacid, statins, signs and symptoms of gastritis and sinusitis were significantly associated with low oral pH levels. However, multiple variable analysis showed, only aspirin was significantly associated with low oral pH levels (odds ratio = 1.91, 95% CI 1.23–2.99, P < 0.01). Duration of aspirin consumption did not show a significant association with low oral pH (P = 0.92). None of the other medications, gastritis or sinusitis were significantly associated with low oral pH levels (Table 3).

**Table 3:** Results of individual variable analysis and multiple variable analysis

Variable	Parameter estimate	Standard error	Z value	P value
Results of individual variable analysis				
Age	− 0.017	0.012	− 1.46	0.14
Gender	− 0.112	0.218	− 0.51	0.61
Aspirin	0.649	0.225	2.89	< 0.01
Aspirin duration	0.0002	0.002	0.10	0.92
Antacids	− 0.542	0.220	− 2.46	0.01
Antihypertensives	0.173	0.219	0.79	0.43
Statins	0.439	0.223	1.97	0.05
Nitrates	− 0.143	0.215	− 0.66	0.51
Gastritis	− 0.475	0.220	− 2.157	0.03
Sinusitis	− 0.570	0.222	− 2.574	0.01
Final model after multiple variable analysis				
Intercept	0.16	0.18	0.88	0.37
Aspirin	0.65	0.23	2.88	< 0.01

Among the 78 patients those who were followed up for 4 weeks with famotidine 20 mg twice per day, 1 had oral pH below 5.5, 30 showed oral pH of 5.5–6.0 and 47 showed oral pH of 6.0–6.5 at the beginning of the follow up period. The patient who had oral pH below 5.5 did not show improvement and from the rest, 63 (81%) showed an improvement in oral pH levels after the follow up period. Among those who showed an improvement in oral pH levels, 74% (i.e. 23 out of 30) were from the group who had oral pH between 5.5–6.0 and 48.9% (i.e. 23 out of 47) were from the group who had oral pH between 6.0–6.5. The number of patients who showed an improvement during the follow up period was significantly higher in the group who had pH levels between 5.5–6.0 compared to those who had a pH level between 6.0–6.5 (Persons’ Chi square test, χ^2^ = 4.7, degree of freedom = 1, P = 0.03).

### Discussion

Our study showed that 69% of aspirin taking patients had oral pH levels less than 6.5 and aspirin showed a significant association with low oral pH levels irrespective of the duration of aspirin prescription. Further, the oral pH level had improved after prescribing an antacid for 4 weeks and the pH improvement was greater in those who had lesser oral pH levels. Since long term tooth exposure to low pH levels leads to demineralisation, our study supports that aspirin taking patients are more prone to experience dental erosions [4]. Although Sullivan and Kramer had showed that swallowed aspirin was not associated with dental erosions in children, and it is possible that there is less exposure time to experience visible erosions in children with swallowed aspirin [9]. Therefore, we also can expect a high number of patients with dental erosions in the study group although we had not assessed the patients for dental erosions.

Even though one of the known side effects of aspirin is gastric irritation [16], only 6.5 and 4.1% of the aspirin taking patients showed signs and symptoms of gastritis and were previously prescribed antacids respectively. This may be, either these patients had not experienced gastritis to a level that they could reveal symptoms or may have been advised to discontinue taking aspirin due to its side effects on a previous medical visit. Therefore, it is unlikely to prescribe an antacid to these patients in the regular medical follow up visits. We would like to highlight the benefit of prescribing an antacid along with aspirin to improve oral pH levels as higher salivary pH facilitates enamel remineralization, effectively buffer acids and suppress aciduric bacteria [17].

Interestingly, 94.5 and 98.4% of the non-aspirin taking patients had signs and symptoms of gastritis and sinusitis respectively [18]. However, we could not find any association between sinusitis with oral pH levels even though sinusitis can produce acidic secretions to oral cavity. Although the majority of aspirin taking patients were taking antihypertensives and statins, none of the medicines showed significant association with low oral pH levels.

## Limitations

This study has several limitations. The study was conducted in a resource poor setting and pH papers were used to measure oral pH levels. Although it has been shown that pH papers are a simple and inexpensive method for patient monitoring [19], we could not obtain the exact pH readings as pH papers could only provide pH readings at 0.5 intervals. It is possible that readings were associated with measurement bias as the readings were obtained by matching pH colour codes and the pH reading would have been better if we could use pH meters. Also, identification of gastritis and sinusitis was based on clinical diagnosis and there could be inter investigator variability in diagnosis as well. We could not study the effect of other individual drugs on oral pH as we grouped the medications into antihypertensives, nitrates and statins. It is possible that effect of an individual medicine in a drug group on oral pH could be masked due to the opposite effect of another drug within the same group.

## Abbreviations

CVD: cardiovascular diseases
Aspirin: acetylsalicylic acid
